# Presurgical Use of Hypoxic Mixture for Systemic Perfusion Improvement in Neonates With Complex Congenital Heart Disease: A Systematic Review and Meta-Analysis

**DOI:** 10.7759/cureus.53409

**Published:** 2024-02-01

**Authors:** Delia E Theurel Martín, Jorge L Alvarado Socarras, Edgar F Manrique Hernández, Mónica A Sandoval, Alvaro J Coronado Munoz

**Affiliations:** 1 Neonatology, Fundación Cardiovascular de Colombia, Floridablanca, COL; 2 Epidemiology and Public Health, Fundación Cardiovascular de Colombia, Floridablanca, COL; 3 Pediatric Critical Care Medicine, University of Miami, Coral Gables, USA; 4 Pediatric Critical Care Medicine, Albert Einstein College of Medicine, Bronx, USA

**Keywords:** qp/qs, systemic perfusion, pulmonary over-circulation, hypoxic mixture, congenital heart disease

## Abstract

Oxygen therapy is essential for the survival of preterm babies and critically ill newborns; however, it has the potential to cause harm through hypoxemia or hyperoxemia. Newborns with complex congenital heart diseases (CHD) suffer from oxygen fluctuations due to the disease and its treatments, altering pre and postnatal development. The objective of this study is to evaluate the evidence for using a hypoxic mixture to decrease pulmonary over-circulation and improve systemic perfusion before surgical interventions in newborns with complex CHD that course with pulmonary over-circulation and systemic hypoperfusion. A search was conducted in PubMed, EMBASE, LILACS, Scielo, Taylor and Francis, SAGE, and Science Direct databases from 2000 to 2022 by two independent authors, including articles with hypoxic mixture treatment in observational studies or trials, with pre-treatment and post-treatment measurements in the same patient, or two groups or more comparisons. Six articles were selected, with a total of 75 patients. The primary outcome was improved systemic circulation and decreased pulmonary over-circulation measured directly with Qp/Qs and indirectly with oxygen saturation and cerebral near-infrared spectroscopy (NIRS). In addition, we performed a meta-analysis for oxygen saturation and cerebral NIRS. Oxygen saturation was the value uniformly reported; three studies reported a significantly lower oxygen saturation after the hypoxic mixture. The cerebral NIRS was measured in 4 studies, with inconsistent results. After using the hypoxic mixture, the Qp/Qs calculation was lower in the two studies but was not statistically significant. The meta-analysis for oxygen saturation showed a fixed effect post-hypoxic therapy of -0.7 (-1.06; -0.35), p < 0.001. The meta-analysis of two studies that measured cerebral NIRS did not show a statistically significant difference at 12 and 24 hours.

In conclusion, this is the first systematic review and meta-analysis regarding the pre-operative use of hypoxic gas mixtures for newborns with complex congenital heart disease. Treatment results in lower oxygen saturations, but there is a lack of evidence of improvement in systemic perfusion. The utilization of this therapy is controversial, and better evidence is necessary.

## Introduction and background

Oxygen is the most used therapy in neonatal intensive care units, and it is essential for the survival of preterm babies and critically ill newborns. The risks of hypoxemia or hyperoxemia are widely known, including increased mortality, neurological injury, bronchopulmonary dysplasia, and retinopathy of prematurity [1,2]. Oxygenation can influence neurological development in newborns, mainly in premature infants, where the brain is more susceptible to injury from hypoxia [3]. Newborns with severe congenital heart diseases (CHD), such as univentricular heart diseases, hypoplastic left ventricle syndrome (HLHS), and ductal-dependent obstructive heart diseases, have low systemic oxygenation and perfusion [4-6]. In these patients, the physiological drop of pulmonary vascular resistance leads to an increased competing pulmonary flow, resulting in pulmonary over-circulation, decreased systemic flow, and systemic hypoperfusion [7].

Several medical or surgical treatments attempt to avoid pulmonary over-circulation and shock by increasing the pulmonary circulation's resistance and improving systemic perfusion. These temporary treatments allow the patients to reach maturity for surgical correction or palliation. One method described in the literature and currently used in some centers is using a hypoxic mixture or sub-atmospheric oxygenation, consistent with an inspired fraction of oxygen (FiO_2_) between 15-20%. The expected effect is to induce pulmonary vasoconstriction, decrease pulmonary circulation, and improve systemic circulation [8-12]. A Pulmonary-Systemic flow ratio (Qp/Qs ratio) calculation is needed to determine the degree of the circulation balance for these patients [4,6]. The normal Qp/Qs ratio is 1:1, but in patients with an unbalanced circulation, it is usually higher than a 3:1 ratio. However, an exact calculation without invasive procedures is not possible. Therefore, peripheral oxygen saturations are used as a surrogate to estimate the Qp/Qs ratio. Another standard bedside measurement used to evaluate organ perfusion is near-infrared spectroscopy (NIRS) in the brain and kidney. A low value can be a marker of decreased organ perfusion; these numbers should increase as systemic perfusion improves [13-17].

In settings where pediatric cardiovascular surgical teams are readily available, surgical pulmonary artery bands are the preferred method to treat pulmonary over-circulation refractory to medical management (inotropes, diuretics, vasodilators). Even though the hypoxic mixture has fallen out of favor, in some centers, including low and lower-middle-income countries, critical care management for these patients relies on hypercarbia or hypoxic mixtures as a bridge treatment for corrective or palliative surgery. This systematic review seeks to evaluate the evidence for using a hypoxic mixture to decrease the pulmonary over-circulation in patients with complex CHD that course with pulmonary over-circulation and systemic hypoperfusion. The study aims to evaluate the improvement in systemic perfusion, measured by oxygen saturation and cerebral NIRS, in newborns with complex CHD and pulmonary over-circulation treated with a hypoxic mixture during the preoperative phase.

## Review

Material and methods

We performed a systematic review of research examining hypoxic mixtures for medical control of pulmonary over-circulation due to CHD before surgical interventions. The inclusion criteria were observational studies or trials that included patients treated with a hypoxic mixture and had a control group or studies including pre-treatment and post-treatment measurements in the same patients. The age of the patients included was pre-term younger than six weeks of corrected age and term neonates younger than six weeks of chronological age. The hypoxic mixture using nitrogen could vary between 14 to 20%. The studies needed to be published between 2000 to 2022 in English or Spanish. The exclusion criteria included studies without baseline or post-treatment data, in a different language than the specified, case reports or case series, and abstracts. 

Search Strategy and Selection Process

Two independent literature searches were performed in PubMed, EMBASE, LILACS, Scielo, Taylor and Francis, SAGE, and Science Direct databases. The first was carried out until July 26, 2021, and the second was performed until August 1st, 2023, to minimize the gap of missing new publications. A complete list of search terms can be found in Table 1. 

**Table 1:** Search terms for the meta-analysis in PubMed, EMBASE, LILACS, Scielo, Taylor and Francis, SAGE, Science direct databases. ᵠ the search was conducted in English and Spanish.

Database	Search terms
Pubmed	(Effectiveness AND "Heart Defects, Congenital"[Mesh] AND "Infant, Newborn"[Mesh] AND ("nitrox" [Supplementary Concept] OR hypoxic mixture)) (congenital heart disease) AND ((hypoxic mixture) OR (sub-atmospheric) OR nitrox) (pulmonary overcirculation) AND ((hypoxic mixture) OR (sub-atmospheric) OR nitrox) (pulmonary artery band) AND ((hypoxic mixture) OR (sub-atmospheric) OR nitrox) Neonatal AND ((hypoxic mixture) OR (sub-atmospheric) OR nitrox)
EMBASE	'Effectiveness' AND 'Heart Defects, Congenital'/exp AND 'Infant, Newborn'/exp AND 'nitrox'] OR 'hypoxic mixture' ‘congenital heart disease’ AND ‘nitrox’ ‘congenital heart disease’ AND ‘low oxygen mixture’ ‘congenital heart disease’ AND ‘hypoxic mixture’ ‘congenital heart disease’ AND ‘hypoxic’ ‘pulmonary overcirculation’ Pulmonary AND overcirculation Nitrox AND newborn Subatmospheric AND congenital heart disease ‘newborn disease’ AND ‘pulmonary band’
LILACSᵠ	(Effectivenes) AND (Congenital Abnormalities) AND (Heart) AND (Infant, Newborn) AND (nitrox) OR (hypoxic mixture)
Scieloᵠ	(*Effectivenes) AND (Congenital Abnormalities) AND (Heart) AND (Infant, Newborn) AND (nitrox) OR (hypoxic mixture)
Taylor and Francis	[All: effectiveness] AND [All: heart defects, congenital] AND [All: infant, newborn] AND [All: nitrox] OR [All: hypoxic mixture] [Keywords: effectiveness] AND [Keywords: heart defects, congenital] AND [Keywords: infant, newborn] AND [Keywords: nitrox] AND [Keywords: hypoxic mixture]
SAGE	[All effectiveness] AND [All heart defects, congenital] AND [All infant, newborn] AND [All nitrox] OR [All hypoxic mixture]
Science direct	Effectiveness AND Heart Defects, Congenital AND Infant, Newborn AND (Nitrox OR hypoxic mixture)

The articles were independently evaluated by two authors, DETM and JLAS. The articles retrieved from the literature search were screened by title and abstract. The approved articles were assessed in full text. In the cases of disagreement between the two evaluators, the decision was solved by consensus. Articles identified with the snowball methodology were evaluated for their subsequent inclusion. Two independent reviewers, MAS and ACM, evaluated the articles included after the second literature search (August 2023) and agreed on the articles included without further additions. The articles selected were assessed for bias and level of evidence by two authors. We utilized The Risk Of Bias In Non-randomized Studies - of Interventions (ROBINS-I) tool for bias assessment. For the level of evidence, we used the GRADE guidelines. The results from these assessments are presented in Table 2. 

**Table 2:** Bias assessment and level of evidence score. First 8 columns are the bias assessment from the ROBINS-I tool. The last column is the level of evidence using the GRADE scale. The third column term NA refers to non-applicable because all the patients were their own control, no possibility of that risk.

First Author	Bias due to confounding	Bias in selection of participants into the study	Bias in classification of interventions	Bias due to deviations from intended interventions	Bias due to missing data	Bias in measurement of outcomes	Bias in selection of the reported result	Overall bias	GRADE
Takami et al., 2005. [16]	Low	Low	Low	High	Low	High	Moderate	Low	Low ꚚꚚOO
Thomas et al., 2019. [17]	Low	Low	Low	Low	Low	Low	Low	Low	Moderate ꚚꚚꚚO
Toiyama et al. 2010. [18]	Moderate	Low	Low	Low	Low	Low	Moderate	Low	Low ꚚꚚOO
Tan et al., 2005. [10]	Low	Low	Low	Low	Low	Low	Low	Low	Low ꚚꚚOO
Ramamoorthy et al. 2002. [8]	Moderate	Low	Low	Low	Low	Low	Moderate	Low	Low ꚚꚚOO
Tabbutt et al., 2001. [9]	Low	Low	Low	Low	Low	Low	Low	Low	Low ꚚꚚOO

Outcomes and Additional Variables

The primary outcome was decreased pulmonary over-circulation and improved systemic perfusion. We used three independent physiological measurements to evaluate this outcome: peripheral oxygen saturation, the brain NIRS, and the Qp/Qs value. At least one of these calculations needed to be available to be included in the analysis. All these measurements were required to be present before and after the hypoxic gas administration in studies where the measurements were done in the same patients. We included the last value reported in our results table in studies with multiple measurements. The data extracted were tabulated and presented in results, including patients' characteristics, interventions, and measurements for the primary outcome.

We collected the data and reported the results of other variables that could alter pulmonary or systemic circulation. These variables include lactate, temperature, vasopressor utilization, pH, carbon dioxide values, arterial pressure of oxygen, and systemic blood pressure.

Statistical Analysis

We conducted a secondary meta-analysis for continuous outcomes (oxygen saturation and cerebral NIRS), reporting the standardized mean difference for fixed and random effects and heterogeneity tests. Some papers reported measurements at different times post-intervention. For the oxygen saturation, we utilized the last reported measure. The times when those measurements were obtained, with the intervention as a reference, are presented in the result section. We excluded one study from the meta-analysis for oxygen saturation as the intervention was titrated to reach a prespecified oxygen saturation target. About cerebral NIRS we included two papers with 12- and 24-hour post-intervention measurements. We performed a meta-analysis for each of those time stamps. We excluded two articles from this analysis: one had measurements 10 minutes post-intervention, which is a very short time-lapse to evaluate a change, and the second included the mean cerebral NIRS measure from the median of multiple measurements for each patient obtained for 90 minutes while the FiO2 was being titrated for target saturation. The Qp/Qs measurement method differed between the two studies, so no further analysis was performed. The statistical analysis was conducted in R.

Results

**Figure 1:**
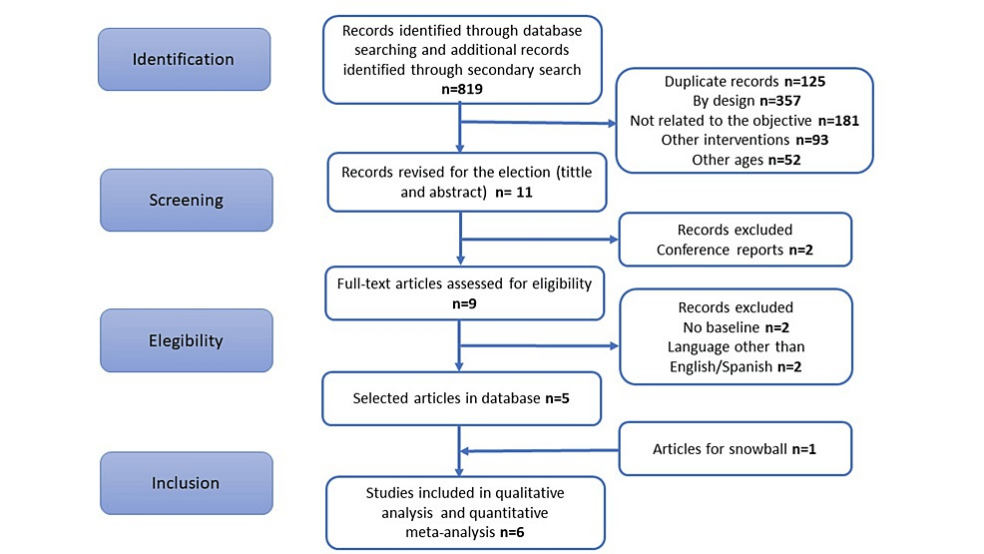
Prisma flowchart of articles search methods and selection.

After completing the full-text review, six studies were included [8-10,16-18]. The initial search retrieved different papers, selecting 11. After reviewing the titles and abstracts, nine were thoroughly examined; of these, two were not included for not having a baseline measurement, one was a letter to the editor, and the last did not include any outcomes of interest. One additional article was found with snowball methodology. The selection is presented in Figure 1. All the studies had measurements before and after the hypoxic mixture treatment with 75 patients in total.

Patients’ Characteristics

All the studies included had neonatal patients; only one study included data exclusively from premature patients. The heart conditions varied from HLHS, tricuspid atresia, interrupted aortic arch, coarctation of the aorta with ventricular septal defect, truncus arteriosus, transposition of great vessels with tricuspid atresia, double inlet left ventricle with hypoplastic right ventricle and AV canal. The study by Tan included premature patients with persistent ductus arteriosus. The study by Toiyama et al. [18] reported that the patients did not have other extracardiac malformations, but the other studies did not report other malformations. All these patient’s characteristics are in Table 3.

**Table 3:** Demographics and clinical characteristics of patients included in each study. Table 3: Demographics and clinical characteristics of patients included in each study. Results in medians with minimum and maximum values in parenthesis. If median was not available mean and standard deviation are reported when specified. For “Heart diagnosis” column in parenthesis is the number of patients for each diagnosis. The abbreviations are: HLHS, hypoplastic left heart syndrome; CoA, coarctation of the aorta; VSD, ventricular septal defect; PDA, patent ductus arteriosus; TGV, transposition of great vessels; TA, tricuspid atresia; DILV, double inlet left ventricle; HRV, hypoplastic right ventricle; AV, atrioventricular; MV, mechanical ventilation; PA, pulmonary artery; NR, not reported; SD, standard deviation; Min, minimum; Max, maximum.

First Author	n	Age (days)	Gestational age (weeks)	Weight (grams)	Heart diagnosis	FiO_2_ goal (%)	FiO_2 _delivery	Treatment duration	Outcome
Takami et al., 2005. [16]	8	Mean 15.9 ± SD 16.8	Mean 36 ± SD 3	Mean 2537 ± SD 576	HLHS (3), Tricuspid atresia (2), Interrupted aortic arch (1), CoA + VSD (1), truncus arteriosus (1)	Mean 16.2 ± SD 1.1	Head box (25%) MV (75%)	Mean 33.4 ± SD 62.6 days	NR
Thomas et al., 2019. [17]	28	Mean 3.5 ± SD 0.8	-	-	Univentricular lesion for Norwood (28)	Mean 17.3 ± SD 1.6	MV	Mean 2.4 ± SD 1.1 days	Norwood (100%)
Toiyama et al. 2010. [18]	8	Mean 6.5 (Min. 0 – Max. 120)	39 (Min. 37 – Max. 42)	3129 (Min. 2816 – Max. 3400)	Tricuspid atresia (2), HLHS (2), Interrupted aortic arch + aortic stenosis (2), CoA + VSD (2)	Mean 17.9 ± SD 1.35	Trans-nasal (75%) MV (25%)	Mean 4.5 ± SD 1.5 days	Norwood (37%) Intracardiac repair (50%) Aortic arch repair + PA banding (13%)
Tan et al., 2005. [10]	6	Mean 14 (Min. 2 – Max. 35)	Mean 26.5 (Min. 25 - Max. 32)	Mean 912 (Min. 706 - Max. 1378)	PDA	18	MV	Mean 40 hours (Min. 35 minutes - Max. 48 hours)	PDA clipping (100%)
Ramamoorthy et al. 2002. [8]	15	Mean 5 ± SD 3	Mean 39 ± SD 2	Mean 3 ± SD 0.6	HLHS (12), TGV+ TA (1) DILV + HRV (1), AV canal (1)	17	MV	10 min	Norwood (100%)
Tabbutt et al., 2001. [9]	10	Mean 5.2 (Min. 1 – Max. 14)	-	Mean 3400 (Min. 2600 - Max. 4000)	HLHS (10)	17	MV	10 min	NR

Hypoxic Mixture Delivery

All the studies included had a hypoxic mixture administered with measurements obtained before and after the intervention. The patients had different times of hypoxic mixture varying from 10 minutes to more than three days, with FiO_2_ values between 14% as a minimum and an average of 17%. The patients had the hypoxic mixture delivered through the mechanical ventilator in 66 (88%) of the patients, 7 (9.5%) were treated transnasally, and 2 (2.5%) with a head box. The patients’ clinical characteristics during the hypoxic mixture are reported in Table 3.

Outcomes

Oxygen saturation was reported in all the studies included. The time of measurement ranged from 10 minutes after treatment to 24 hours. Three studies (Tan et al., Tabbutt et al., and Thomas et al.) had statistically significant differences between baseline and post-hypoxic mixture exposure, one did not report statistical significance, but the value post-hypoxic mixture was 80.8 ± 2.9 from 97 ± 2.1. Two studies did not find statistically significant differences but reported lower levels after the intervention. The paO_2_ was reported in 4 studies, and all found statistically significant lower levels after the hypoxic measurement. The exact values are in Table 4. 

**Table 4:** Primary outcome and physiological parameters measured in the included studies. All measurements reported in mean and standard deviation. In parenthesis is the time when measurement was done. Statistical significance p value <0.05. Abbreviations: NIRS, near-infrared spectroscopy measuring the mixed cerebral vascular oxygen saturation; paO2, arterial partial pressure of oxygen; Qp/Qs, pulmonary blood flow to systemic blood flow ratio; SBP, systolic blood pressure; h, hours; min, minutes; NS, not significant; NR, not reported. *FiO2 titrated to obtain oxygen saturations of 80%. NIRS means are obtained from median values for each patient from multiple measurements over 90 minutes during the FiO2 titration for oxygen saturation goal of 80%. **Measurements at the end of hypoxemic mixture performed at different times for each patient. § Different methods of measurement in two studies reporting Qp/Qs. §§ Values pre and post intervention not reported.

		Oximetry (%)		Oximetry p-value	paO_2_ (mmHg)		paO_2_ p-value	Cerebral NIRS (%)		NIRS p-value	Qp/Qs^§^		Qp/Qs p-value	Systolic pressure (mmHg)		SBP p-value
FiO2 group:		21%	15-21%		21%	15-21%		21%	15-21%		21%	15-21%		21%	15-21%	
Author	n															
Takami et al., 2005. [16] *	8	97 ± 2.1	80.8 ± 2.9 (90 min)	NR				57.6 ± 7.7	49.3 ± 8.5 (90 min)	NR				65 ± 6.5	66.7 ± 8.7	NS
Thomas et al., 2019. [17]	28	91.7 ± 6.6	86.0 ± 6.5 (24h)	<0.05	45.3 ± 8.3	36.3 ± 5.1 (24h)	<0.05	60.1 ± 12.8	58.8 ± 9.9 (24h)	NS	9.6 ± 13.0	3.3 ± 3.6 (24h)	<0.05	65.5 ± 9.7	67.6 ± 8.7 (24h)	NS
Toiyama et al. 2010. [18]	8	91.9±4.6	90.3±4 (12h)	NS				67.3±11.1	70.7±6.7 (24h)	NS						
Tan et al., 2005. [10] **	6	97 ± 4	94 ± 4	0.03	67 ± 19	54 ±15	0.03							44 ± 8.8	55 ± 7	0.03
Ramamoorthy et al. 2002. [8]	15	95±4	94±4 (10 min)	NS	51 ± 7	47 ± 5 (10 min)	<0.01	53 ± 13	53 ± 14 (10 min)	0.85				64 ± 11	61 ± 10 (10 min)	NS
Tabbutt et al., 2001. [9]	10	93.2 ± 1.5	89.8 ± 2.8 (10 min)	0.004	50.3 ± 1.9	42.2 ± 1.8 (10 min)	<0.001	- 0.4 ± 1.5^§§^ (10 min)		0.8	3.36 ± 0.46	2.55 ± 0.48 (10 min)	0.056	35 ± 2.1	33.7 ± 1.9 (10 min)	0.79

Cerebral saturation was reported in four studies that show the comparison between baseline and after hypoxic mixture therapy [8, 16-18]. None of those studies had a statistically significant value decrease or increase (Table 4). The study by Thomas et al. had inconsistent results over time, with lower levels at 6 and 12 hours and higher at 24 hours. The study by Toiyama et al. showed higher levels at 1, 12, and 24 hours. Ramamoorthy et al. reported results after 10 minutes of intervention, showing decreased cerebral saturation in 8 of 15 patients with a maximum difference of 6.5%, one patient did not have a different measurement, and 6 had an increase to a maximum of 7% [8]. In the last study reporting cerebral NIRS, Takami et al. showed a decrease in NIRS with a mean difference of -8.3 ± 2.6. The results from Takami were obtained from the median of repeated values over 90 minutes.

Two studies analyzed reported Qp/Qs measurements at baseline and after the hypoxic mixture (Table 4) [9,17]. The Qp/Qs measurement methods differed between the two studies. Only one study (Thomas et al.) showed a significant decrease in the hypoxic mixture, clustering their calculation at 24 hours but not at 6 and 12 hours. However, this study used renal NIRS to calculate the Qp/Qs. The study by Tabbutt did not show a significant difference, but there was a decrease in the Qp/Qs from 3.36 ± 0.46 to 2.55 ± 0.48, p 0.056. This change occurred ten minutes after the hypoxemic mixture. No other hemodynamic outcomes were reported in the included studies.

Additional Variables of Interest

Four studies reported that the patients had surgery post-treatment, no studies reported complications, and one reported that all their patients were discharged home post-surgically. In the review, four of the six studies reported comparative systolic blood pressure before and after treatment, one of which mentioned a statistically significant increase (Tan et al.). Three of the six studies reported a statistically significant improvement in pH after the hypoxic treatment (Ramamoorthy et al., Tabbutt et al., and Tan et al.). The study by Tan et al. reported a pH improvement from 7.29±0.21 to 7.34±0.12, p 0.03. The study by Takami et al. reported a balanced pH at the time of the intervention, i.e. 7.44±0.003, and a stable PaCO_2_ at 43.2 ± 4.9 mmHg after the hypoxic mixture. One study reported lower carbon dioxide levels after the hypoxic mixture without alteration in minute ventilation (Ramamoorthy et al.). The study by Tabutt et al. reported a lower PCO2 after the hypoxic mixture, 37.5 ± 1.8 vs. 35.7 ± 2 mmHg. Toiyama et al. reported no deterioration in pH, pCO2, pH, systemic blood pressure, or lactic acid levels. One study measured regional saturation at the renal level (RrSO2) to assess perfusion and systemic oxygenation, which showed non-statistically significant lower measurements after the hypoxic mixture (Thomas et al.). The study made by Tabutt et al. reported mixed central venous saturation and the calculation of oxygen delivery, both with a non-statistically significant decrease after the hypoxic mixture. The study made by Thomas et al. reported a non-statistically significant reduction in lactate.

Meta-analysis

**Figure 2:**
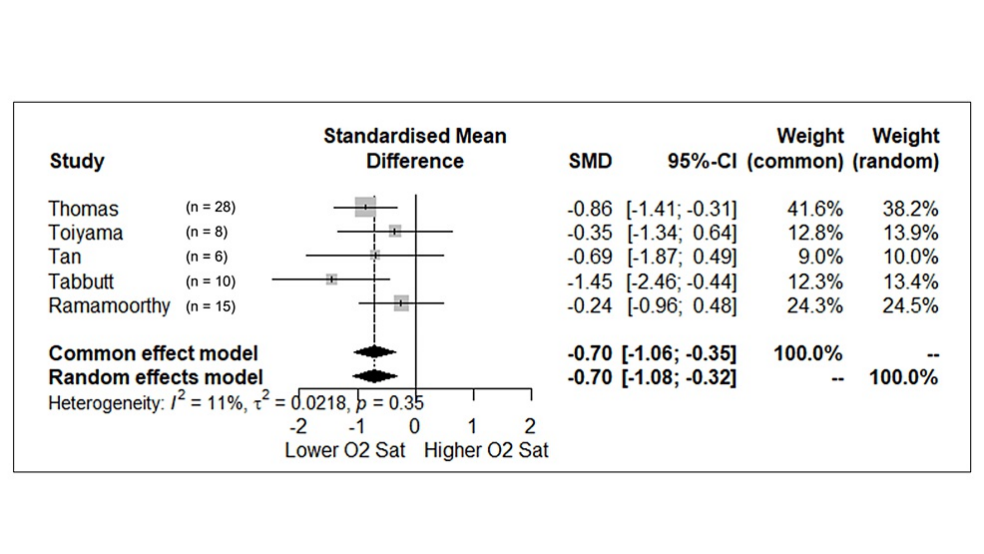
Forest Plot of oxygen saturation. SMD, standardized mean difference. Ramamoorthy et al. 2002 [8]. Tabbutt et al., 2001 [9]. Tan et al., 2005 [10]. Thomas et al., 2019 [17]. Toiyama et al. 2010 [18].

We performed a meta-analysis for oxygen saturation and NIRS. The meta-analysis of the post-intervention oxygen saturation of five studies showed a standardized mean difference and 95% CI for the fixed effect of -0.7 (-1.06; -0.35), p < 0.001. This result has an I2 of 11%, τ2 of 0.02, and a Cochran’s Q test of 0.35, indicating no heterogeneity. The results are presented in a Forest plot in Figure 2. One study (Takami et al.) titrated down the FiO_2_ until the patient reached an oxygen saturation goal of 80%. (Table 4) Due to that methodology and the fact that oxygen saturation was a condition created with a predetermined value, we did not include that study in the meta-analysis for oxygen saturation. For NIRS, only the two studies with measurements at the same time were included. We performed two meta-analyses, with the first two studies (Thomas and Toiyama) utilizing the measurements at 12 and 24 hours. The results did not show a significant difference, and both had low heterogeneity. The forest plots for these analyses are in Figure 3 in the supplement material. 

**Figure 3:**
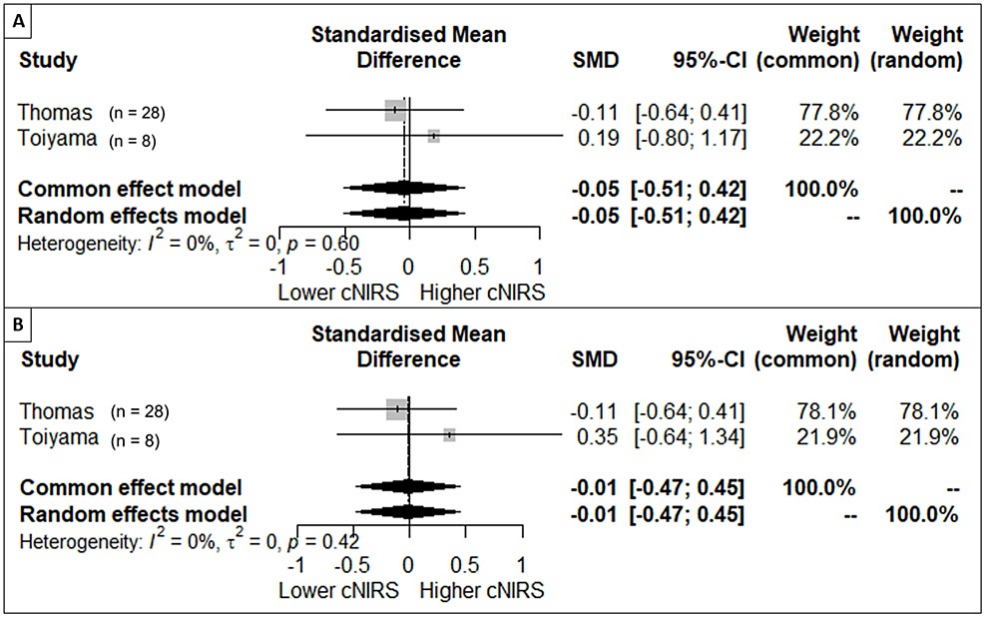
Forest Plot of cerebral NIRS (cNIRS). Panel (A) at 12 hours; Panel (B) at 24 hours. SMD, standardized mean difference. Thomas et al., 2019 [17]. Toiyama et al. 2010 [18].

Discussion

We examined the evidence available for this therapy to demonstrate if it helps improve perfusion. The included articles measured different parameters, but the evidence for perfusion improvement was limited and inconclusive. We included six studies in this systematic review and meta-analysis showing that patients treated with a hypoxic mixture had a lower oxygen saturation after this therapy. However, we did not find a statistically significant difference in the cerebral NIRS, and we could not include Qp/Qs because of the different measurement methods used in the studies reporting it. This study demonstrates the lack of evidence and protocols to use this therapy.

Oxygen saturation was the value uniformly reported in the included studies. Most studies reported lower oxygen saturation after the hypoxic mixture, and our meta-analysis found a statistically significant difference. A higher oxygen saturation than expected for these patients is usually a surrogate of high pulmonary flow. However, lower saturation may not always reflect a balanced Qp/Qs ratio. Most complex CHD infants are systemically hypoxemic, a greater decrease in oximetry, without evidence of improved tissue oxygenation, could simply indicate pulmonary desaturation. This is subject to interpretation, and obtaining other measurements to determine systemic output, like mixed venous saturation or Doppler aortic flow, is necessary to decide the correct intervention [7, 19, 20]. The studies that reported a lower oxygen saturation during the treatment with a hypoxic mixture did not report shock or signs of decreased systemic perfusion. Currently, the measurement of continuous regional tissue oxygenation through NIRS is frequently utilized. The results of the studies that reported cerebral NIRS involved different FiO2 during the hypoxic mixtures, with one showing different variations over time, one showing slight improvement, and one showing worsening. The cerebral NIRS values with a hypoxic mixture could be directly related to the lower oxygenation and are not only a marker of low perfusion [20]. The best possible outcome to assess the effect of hypoxic gas in this population is the Qp/Qs ratio. The relationship of hypoxia and hypercarbia in the Qp/Qs balance for this specific population was described by Barnea et al. [7]. The exact measurement of the Qp/Qs can only be possible with invasive procedures. We could not determine a significant improvement of Qp/Qs with the information from the two studies that reported it, both with a Qp/Qs improvement but with different measurement methods. The additional measurements included in these studies were non-contributory, and some were not reported. These measurements included additional mediators of pulmonary vasoactivity like PaCO_2_, systemic blood pressure, patient temperature, and pH. Most studies reported these levels as unchanged during the hypoxic mixture treatment.

The management of patients with competing parallel circulations is challenging. The definitive treatment is not always possible upon diagnosis. It is vital to know how to temporize the medical management of these patients until a surgical option becomes available. Our study shows that there is no data to support or be against the hypoxic mixture treatment. Dropping the oxygen saturation without improving perfusion can worsen any state of shock. The studies presented are pre- and post-measurements with this therapy in the same subjects, lacking randomization and with significant inconsistencies in the time and mode of delivery of the therapy. Additionally, no trials or cohort studies exist comparing pulmonary artery banding (PAB) to medical management, and within medical options, there are no studies comparing hypercarbia and hypoxic mixture [9,12,21-25]. It is unknown what is a safe length of therapy with a hypoxic mixture. In two of the studies, they used a hypoxic mixture for more than three days without reported complications. Other medical treatments, like optimizing inotropic support, systemic vasodilators, adequate ventilation with ideal PEEP, maintaining adequate urine output, and optimal hemoglobin, must be included in the current management of these patients [12]. Given that the hypoxic mixture will decrease oxygen saturation, the decision to initiate this therapy should be preferably made with an appropriate assessment of the cardiac output. A group of authors also proposed to evaluate the risk of pulmonary over-circulation using pulmonary ultrasonography, such as the LUCAS score (Lung Ultrasonography in Cardiac Surgery) [26]. Evaluating the impact of medical management and PAB directly on lung circulation could be a relevant outcome to measure in future studies. The safety of using a hypoxic mixture has yet to be established.

Severe CHD exposes newborns to lower oxygen levels, compromising cerebral oxygenation and altering pre and postnatal brain development [27-29]. When investigating this topic, we were interested in finding evidence proving whether this therapy is safe for brain perfusion or poses a risk. Exposure to hypoxic mixtures and administering lower than physiological oxygen concentration could be an added risk for neurological injury. Unfortunately, none of the studies discusses a total and comprehensive neurological evaluation during hospitalization or external neurodevelopment follow-up of patients who had hypoxic mixture therapy. More extensive investigations, including the pre-surgical management of these patients, looking over time from birth to surgical correction and neurological outcomes, are necessary, including any confounders that could impair the systemic perfusion.

Our study has limitations on the type of studies available for review and meta-analysis. As presented in Table 2, the risk of bias overall is low, and the GRADE quality of evidence is low for all the studies, except Thomas et al., which was qualified as moderate due to the number and the equal diagnosis of the included patients. As discussed, all the studies were pre- and post- with different methods of hypoxic mixture administration and the lack of information regarding tissue oxygenation (cardiac output, hemoglobin). Most studies incorporated single ventricle physiology diagnosis except Tan et al., which presented preterm newborns with PDA and highly unbalanced circulation. Other important limitations are the scarce publications about the topic, the small sample size of the assessed studies, and the lack of information regarding the management before and during the intervention (inotropes, prostaglandin, diuretics, saline boluses, sedation, red blood cells, and others), the different variables for measurement of O_2_ delivery between each study, and the lack of uniform outcomes, specifically to perform a more comprehensive metanalysis.

## Conclusions

This study is the first systematic review and meta-analysis regarding the pre-operative use of hypoxic gas mixtures to avoid pulmonary overcirculation and improve systemic perfusion in newborns with complex CHD and competing circulations. Treatment with hypoxic mixtures in these patients results in lower oxygen saturations. However, there is not enough information regarding improvement in systemic perfusion during its use. Further research is needed to determine the safety and efficacy of this therapy and provide better evidence, recommendations, and protocols for its use.
